# Late-onset *cblC* deficiency around puberty: a retrospective study of the clinical characteristics, diagnosis, and treatment

**DOI:** 10.1186/s13023-022-02471-x

**Published:** 2022-09-02

**Authors:** Zhehui Chen, Hui Dong, Yupeng Liu, Ruxuan He, Jinqing Song, Ying Jin, Mengqiu Li, Yi Liu, Xueqin Liu, Hui Yan, Jianguang Qi, Fang Wang, Huijie Xiao, Hong Zheng, Lulu Kang, Dongxiao Li, Yao Zhang, Yanling Yang

**Affiliations:** 1 Department of Pediatrics, Peking University First Hospital, Beijing, 100034 China; 2 Department of Pediatrics, Peking University People’s Hospital, Beijing, 100034 China; 3 Department of Respiratory Medicine II, Beijing Children’s Hospital Affiliated to Capital Medical University, Beijing, 100045 China; 4 Department of Clinical Laboratory, China-Japan Friendship Hospital, Beijing, 100029 China; 5 Department of Pediatrics, First Affiliated Hospital of Henan University of Traditional Chinese Medicine, Zhengzhou, 450000 China; 6 Department of Pediatrics, The First Affiliated Hospital of Zhengzhou University, Zhengzhou, 450052 China; 7 Henan Key Laboratory of Children’s Genetics and Metabolic Diseases, Children’s Hospital Affiliated to Zhengzhou University, Zhengzhou, 450053 China

**Keywords:** Methylmalonic aciduria, cblC, Adolescence, Puberty, Neuropsychiatric symptoms, Multiple organ damage

## Abstract

**Background:**

cblC deficiency is the most common type of methylmalonic aciduria in China. Late-onset patients present with various non-specific symptoms and are usually misdiagnosed. The purpose of this study is to investigate the clinical features of patients with late-onset cblC deficiency and explore diagnosis and management strategies around puberty.

**Results:**

This study included 56 patients (35 males and 21 females) with late-onset cblC deficiency who were admitted to our clinic between 2002 and September 2021. The diagnosis was confirmed by metabolic and genetic tests. The clinical and biochemical features, disease triggers, outcome, and associated genetic variants were examined. The onset age ranged from 10 to 20 years (median age, 12 years). Fifteen patients (26.8%) presented with symptoms after infection or sports training. Further, 46 patients (82.1%) had neuropsychiatric diseases; 11 patients (19.6%), cardiovascular diseases; and 6 patients (10.7%), pulmonary hypertension. Renal damage was observed in 6 cases (10.7%). Genetic analysis revealed 21 variants of the *MMACHC gene in the 56* patients. The top five common variants detected in 112 alleles were c.482G > A (36.6%), c.609G > A (16.1%), c.658_660delAAG (9.8%), c.80A > G (8.0%), and c.567dupT (6.3%). Thirty-nine patients carried the c.482G > A variant. Among 13 patients who exhibited spastic paraplegia as the main manifestation, 11 patients carried c.482G > A variants. Six patients who presented with psychotic disorders and spastic paraplegia had compound heterozygotic c.482G > A and other variants. All the patients showed improvement after metabolic treatment with cobalamin, l-carnitine, and betaine, and 30 school-aged patients returned to school. Two female patients got married and had healthy babies.

**Conclusions:**

Patients with late-onset cblC deficiency present with a wide variety of neuropsychiatric symptoms and other presentations, including multiple organ damage. As a result, cb1C deficiency can easily be misdiagnosed as other conditions. Metabolic and genetic studies are important for accurate diagnosis, and metabolic treatment with cobalamin, l-carnitine, and betaine appears to be beneficial.

**Supplementary Information:**

The online version contains supplementary material available at 10.1186/s13023-022-02471-x.

## Background

Puberty is a critical stage for manifestation of late-onset inherited metabolic disorders. cblC deficiency is the most common defect in the intracellular cobalamin metabolism pathway and is characterized by variable and non-specific symptoms [1, 2]. Methylmalonic aciduria (MMA) combined with homocystinuria caused by cblC deficiency (OMIM 277400) accounts for 70% of the cases of MMA, which is the most common organic acid metabolic disorder in China [3–5]. The prevalence of cb1C deficiency in Shandong province of China was 1/3,920 according to data of Newborn Screening from 2011 to 2014 [6], but the nationwide prevalence of cblC deficiency is unclear.

In patients with cblC deficiency, the age of onset ranges from the prenatal to adult stage, and the clinical manifestations vary from mild to life-threatening [1, 2, 7]. The most common phenotype is the early-onset type, which usually affects the nervous system and mainly presents with developmental delay, epilepsy, lethargy, and hypotonia. In addition, the disease is often complicated with multiple organ damage, such as visual impairment, renal damage, hematological abnormalities, and cardiovascular diseases. Neonatal-onset cb1C deficiency is associated with critical illness, rapid progression, and high mortality. In the case of infancy-onset cb1C deficiency, infection and starvation are the most common triggers of metabolic crisis. Infants usually present with developmental delays, seizures, and confusion [2, 6]. Adolescence is a high-risk period for the manifestation of cblC deficiency. Most teenagers with late-onset cblC deficiency develop behavioral abnormalities, mental regression, or movement disorders [5, 8]. These symptoms are non-specific, and patients are easily misdiagnosed. Recently, more cases of late-onset cblC deficiency have been diagnosed, with some cases being reported in adolescence or adulthood [7, 9, 10]. Plasma total homocysteine (tHCY), blood amino acid levels, acyl-carnitine analysis, and genetic study are important for obtaining a definite diagnosis [11], and patients usually recover after receiving metabolic treatment with hydroxycobalamin or adenosylcobalamin, l-carnitine, and betaine [8, 11, 12]. However, most late-onset patients exhibit non-specific symptoms that may lead to misdiagnosis and mismanagement.

In our previous study on 1,003 patients with MMA, 705 (70.3%) had MMA combined with homocystinemia caused by cb1C deficiency and 567 (80.2%) had early-onset disease (before the age of 1 year). A total of 51 patients (7.2%) had late-onset disease (after the age of 4 years) and showed significant differences in phenotypes and outcomes [4]. However, there is not enough research on the clinical presentations, diagnosis, or treatment of late-onset cb1C deficiency. Therefore, in the present study, we have investigated the clinical features, triggers, metabolic profiles, and genotypes of late-onset cblC deficiency to gain an understanding of the early interventions that may be beneficial to treat and reverse this disease.

## Results

### Clinical course

#### Age of onset

The present study cohort included 56 patients, including 35 (62.5%) males and 21 females (37.5%) (Table 1). The age of onset ranged from 10 to 20 years (median age, 12 years). A total of 31 patients (55.4%) presented with symptoms between the ages of 10 and 12 years. Fifteen patients (26.8%) presented with symptoms within the age range of 13–15 years. Five patients (8.9%) developed symptoms between the ages of 16–18 years. Five patients (8.9%) presented with symptoms in the age range of 18–20 years.

**Table 1:** Clinical manifestations of 56 patients with late-onset cblC deficiency around puberty

Clinical manifestations	Age of onset										Blood marker levels before treatment		
	10–12 years		13–15 years		16–18 years		19–20 years		Total		tHcy (µMol/L)	C0 (µMol/L)	C3 (µMol/L)
	n	%	n	%	n	%	n	%	n	%			
Neuropsychiatric diseases	24	42.9	12	21.4	4	7.1	5	8.9	45	80.4	91.5–205.9	6.5–19.5	2.0–14.7
Movement disorders	17	30.4	10	17.9	2	3.6	4	7.1	33	58.9			
Psychotic behavior disorders	13	23.2	2	3.6	4	7.1	2	3.6	21	37.5			
Mental regression	11	19.6	3	5.4	1	1.8	3	5.4	18	32.1			
Seizures	6	10.7	4	7.1	1	1.8	3	5.4	14	25.0			
Spastic paralysis	5	8.9	5	8.9	1	1.8	2	3.6	13	23.2			
Visual impairments	0	0	1	1.8	1	1.8	0	0	2	3.6			
Lethargy/coma	1	1.8	4	7.1	0	0.0	1	1.8	6	10.7			
Cardiovascular disease	4	7.1	6	10.7	0	0.0	0	0	10	17.9	66.3–213.8	7.6–16.6	2.9–11.1
Pulmonary hypertension	2	3.6	3	5.4	0	0.0	0	0	5	8.9			
Hypertension	2	3.6	1	1.8	0	0.0	0	0	3	5.4			
Cardiomyopathy	1	1.8	1	1.8	0	0.0	0	0	2	3.6			
Thrombus	0	0	1	1.8	0	0.0	0	0	1	1.8			
Arrythmia	0	0	2	3.6	0	0.0	0	0	2	3.6			
Renal damage	2	3.6	4	7.1	0	0.0	0	0	6	10.7	48.3–179.7	29.3–38.2	4.5–9.5
Proteinuria	2	3.6	4	7.1	0	0.0	0	0	6	10.7			
Renal insufficiency	1	1.8	0	0	0	0.0	0	0	1	1.8			
Others	10	17.9	5	8.9	2	3.6	0	0	17	30.4	48.3–213.8	5.6–17.5	2.8–11.1
Anemia	4	7.1	4	7.1	0	0.0	0	0	8	14.3			
Anorexia	3	5.4	0	0	1	1.8	0	0	4	7.1			
Obesity	3	5.4	1	1.8	0	0.0	0	0	4	7.1			
Fatty liver	0	0	0	0	1	1.8	0	0	1	1.8			
Visual impairment	0	0	2	3.5	0	0	0	0	2	3.5	163	29.3	4.5
Total	31	55.4	15	26.8	5	8.9	5	8.9	56	100.0			

*n* number; *tHcy* total homocysteine; *C0* free carnitine; *C3* propionylcarnitine

#### Precipitating factors

Eight patients (14.3%) presented with symptoms on the day of fever or several days after infection, and the initial diagnosis in these cases was pneumonia or encephalitis. Seven patients (12.5%) presented with neuropsychiatric symptoms, such as psychotic behavioral disorders and movement disorders, on the day of strenuous exercise or several days after a sports training program. These patients were transitioning from primary school to middle school or from middle school to high school. The parents declared that the children did not have neurological or psychiatric problems prior to disease onset.

#### Symptoms

Forty-five patients (80.4%) mainly presented with neuropsychiatric diseases, and 33 patients (58.9%) had movement disorders. Thirteen patients (23.2%) presented with progressive spastic paralysis, and 21 patients (37.5%) had psychotic behavioral disorders, such as short temper, speaking nonsense words, hallucination, apathy, and overeating. Further, 18 patients (32.1%) showed signs of mental regression, such as memory loss, study weariness, and decrease in grade.

Ten patients (17.9%) mainly presented with cardiovascular diseases, and five patients (8.9%), with pulmonary hypertension. Cardiomyopathy was found in 2 patients (3.6%), and 2 patients (3.6%) had arrhythmia. Further, 6 patients (10.7%) presented with proteinuria, and 1 patient developed renal insufficiency.

8 (14.3%) patients had anemia; 4 (7.1%), anorexia; 4 patients (7.1%), overeating and obesity; 1 patient (1.8%), fatty liver; 2 patients (3.5%), visual impairments (nearsightedness, strabismus, and astigmatism) (Table 1).

#### Misdiagnosis

Prior to being diagnosed with cblC deficiency, the 56 patients had been misdiagnosed with other diseases and had received inappropriate treatment for 2 months to 6 years. The initial diagnosis was peripheral neuropathy, depression, schizophrenia, encephalitis, primary pulmonary hypertension, and epilepsy. Eight patients (14.3%) were misdiagnosed with encephalitis. Their manifestations included mental regression (3 cases, 37.5%), depression (4 cases, 50.0%), and behavioral abnormalities (5 cases 62.5%). Five patients (8.9%) exhibited proteinuria and/or hematuria during routine urine tests and had been previously diagnosed with nephritis.

### Biochemical findings

All the patients had abnormal blood amino acid and acyl-carnitine profiles before treatment (Table 1). Their plasma tHCY values (48.3–213.8 mol/L; normal control value, < 15.0 mol/L) were significantly increased. In addition, elevated blood propionyl-carnitine (2.0–14.7 mol/L; normal control values, < 5.0 mol/L), propionyl-carnitine/acetyl-carnitine ratios (0.58–0.97; normal control value, < 0.5), and propionyl-carnitine/free carnitine ratios (0.3–0.74; normal control, < 0.25) were observed. Further, 12 patients had decreased blood free carnitine levels (5.55–14.51 mol/L; normal control range, 15.0–60.0 mol/L), and 40 patients had decreased blood methionine levels (5.4–9.5 mol/L; normal control range, 12.0–50.0 mol/L). Urine methylmalonic acid concentrations in all the patients were elevated (53.1–1787.0 mmol/mol creatinine; normal control range, 0.2–3.6 mmol/mol creatinine). These biochemical findings supported a diagnosis of MMA combined with homocystinuria. In addition, decreased serum 25-OH-vitamin D levels were observed in 15 patients (26.8%).

### Genetic features

Twenty-one reported pathogenic variants were detected in the *MMACHC* gene of the 56 patients (Table 2, Additional file 1), and one patient got a *PRDX1* variant causing secondary epimutation in *MMACHC* (*PRDX1* c.515-48_515-47 insTT). The top five common variants were c.482G > A (36.6%), c.609G > A (16.1%), c.658_660delAAG (9.8%), c.80A > G (8.0%), and c.567dupT (6.3%). c.482G > A was the most frequent variant and was identified in 41 (36.6%) alleles. Further, 39 patients with neuropsychiatric diseases were found to have at least one allele mutated in c.482G > A. Two cases were homozygous for c.482G > A. Of 13 patients who presented with spastic paraplegia as the main manifestation, 11 had c.482G > A (84.6%). Six patients presented with both psychotic disorders and spastic paraplegia, and all of them had c.482G > A. Ten patients had compound heterozygotic variants of c.482G > A and c.658_660delAAG; seven patients displayed compound heterozygotic variants of c.482G > A and c.609G > A; and five patients had compound heterozygotic variants of c.482G > A and c.567dupT. c.609G > A was the second most common variant observed in the patients and was found in 18 cases (16.1%). Eleven patients had a c.609G > A variant and presented with neuropsychiatric diseases. Seven patients with c.80A > G and c.609G > A compound heterozygotic variants had neuropsychiatric symptoms and pulmonary hypertension.

**Table 2:** *MMACHC* variants in 112 alleles of 56 patients with late-onset cb1C deficiency

*MMACHC* gene variants*					Phenotypes								PMID	References
No.	Nucleotide change	Protein change	n	%	Neuropsychiatric diseases		Cardiovascular diseases		Renal diseases		Others			
					n	%	n	%	n	%	n	%		
1	c.482G > A	p.R161Q	41	36.6	39	34.8	3	2.7	1	0.9	11	9.8	20631720	[3]
2	c.609G > A	p.Trp203Ter	18	16.1	11	9.8	7	6.3	5	4.5	7	6.3	20631720	[3]
3	c.658_660delAAG	p.K220del	11	9.8	11	9.8	1	0.9	0	0	1	0.9	20631720	[3]
4	c.80A > G	p.Gln27Arg	9	8.0	2	1.8	8	7.1	4	3.6	4	3.6	20631720	[3]
5	c.567dupT	P.Ile190Tyrfs*13	7	6.3	7	6.3	0	0	0	0	1	0.9	30157807	[31]
6	c.394C > T	p.Arg132Ter	4	3.6	4	3.6	0	0	0	0	2	1.8	20631720	[3]
7	c.315C > G	p.Tyr105Term	2	1.8	2	1.8	1	0.9	0	0	2	1.8	20631720	[3]
8	c.217C > T	p.Arg73Term	2	1.8	1	0.9	0	0	0	0	2	1.8	16311595	[20]
9	c.615C > A	p.Tyr205Ter	2	1.8	2	1.8	0	0	0	0	1	0.9	20631720	[3]
10	c.444_445delTG/c.445_446delTG	p.C149Hfs*32	2	1.8	2	1.8	0	0	1	0.9	0	0	32943488	[22]
11	c.365A > T	p.His122Leu	2	1.8	1	0.9	1	0.9	0	0	0	0	20631720	[3]
12	Exon 1 del	Exon 1 deletion	2	1.8	2	1.8	0	0	0	0	1	0.9	31278756	[32]
13	c.626dup	p.Thr210fs	1	0.9	0	0	1	0.9	0	0	1	0.9	20631720	[3]
14	c.452A > G	p.His151Arg	1	0.9	1	0.9	0	0	0	0	0	0	20631720	[3]
15	c.656_658delAGA	p.K220Rfs*71	1	0.9	1	0.9	0	0	0	0	0	0	30863077	[5]
16	c.427C > T	p.Gln143Ter	1	0.9	1	0.9	0	0	0	0	0	0	32943488	[22]
17	c.467G > A	p.Gly156ASp	1	0.9	1	0.9	0	0	0	0	0	0	16311595	[20]
18	c.637G > T	p.Glu637Ter	1	0.9	1	0.9	0	0	0	0	0	0	30157807	[31]
19	c.565C > T	p.Arg189Cys	1	0.9	1	0.9	0	0	0	0	0	0	31279840	[19]
20	c.347T > C	p.Leu116Pro	1	0.9	1	0.9	0	0	0	0	0	0	16311595	[20]
21	c.600G > A	p.Trp200Term	1	0.9	1	0.9	0	0	0	0	0	0	16311595	[20]
Total			111	99.1										

*The reference for the transcripts was NM_015506.2. 112 alleles were involved in 56 patients. One patient had a c.609G > A variant **i**n *MMACHC* and c.515-48_515-47insTT in *PRDX1*

### Long-term treatment and follow-up

All 56 patients were treated with intramuscular injection of hydroxycobalamin (1 mg or 10 mg, two or three times a week) or adenosylcobalamin (1.5 or 3 mg, two or three times a week), supplemented with oral l-carnitine (1–2 g/d), betaine (3–6 g/d), and normal diet. For the patients with vitamin D deficiency, oral vitamin D supplementation was also recommended. All the patients showed significant clinical improvement after the metabolic treatment. Follow-up sessions were scheduled at 3, 6, and 12 months. Currently, the patients are 12–32 years old. Among the 33 patients who had movement disorders during the acute phase of the disease, 30 have recovered. The remaining three patients still have an unsteady walk because of spastic paralysis. Among the 30 patients with mental regression or psychotic problems, 29 have recovered and returned to school. One female patient is emotionally unstable and refuses to go back to school. Of the 12 patients who have reached adulthood, nine of them have successfully graduated from college and are working. Further, two female patients got successful pregnancies and had healthy babies [13].

## Discussion

In the present study, we have described the clinical course, biochemical features, genetic findings, and the outcomes of 56 late-onset patients with combined MMA and homocystinuria caused by cblC deficiency. As there is a very little research on this topic, our findings will be valuable for both clinicians and physicians who encounter such cases in their settings.

In the present study, 56 previously healthy school children presented with varied manifestations of cb1C deficiency during adolescence. The parents declared that the children exhibited normal development without any neurological or psychiatric problems and other diseases before onset. Infection and strenuous exercise were considered to be the triggers in 15 patients who presented with movement disorders or psychotic symptoms after having infection or fatigue. Intense exercise and stress can trigger underlying metabolic diseases, so it is important to carefully investigate potential diseases in school children with exercise intolerance or training-related illnesses. Since these symptoms were non-specific, the present patients were misdiagnosed with schizophrenia, depression, autoimmune encephalitis, or neuromuscular diseases. Blood tHCY, amino acids, and acyl-carnitine profiles, and genetic analysis are important for the definite diagnosis of cblC deficiency and must be considered in previously healthy adolescents who present with such symptoms.

In the present study, 45 patients (80.4%) had neuropsychiatric symptoms and 33 patients had movement disorders. In addition, 21 patients had psychotic behavior disorders, such as bad temper, speaking nonsense word, and hallucinations, and 18 patients exhibited symptoms of mental regression (for example, their parents reported sudden onsets of inability to learn). A recently published China Mental Health Survey showed that depressive disorders have a high prevalence in adolescence [14], late-onset cblC deficiency around puberty maybe one of the causes. Five patients in the cohort presented with pulmonary hypertension, with the majority of the complaints being intolerance to sports or fainting during sport activities. Proteinuria and anemia were observed in some cases. All these findings were correlated with previously published literature [15–17]. Seventeen patients (29.8%) presented with other complications. Eight cases had anemia in the acute phase of the disease that improved quickly after metabolic treatment. Overeating and obesity were observed in four cases, and the patients’ physique improved gradually with the improvement of their mental symptoms. Nearsightedness, strabismus, and astigmatism were observed in two patients. However, these are common visual impairments in the general population, and it is difficult to determine whether they are related to cblC deficiency in the two cases.

Genetic study is crucial for a definite diagnosis of cblC deficiency. The mutation spectrums of the *MMACHC* gene vary among different populations [18, 19]. Among the 56 patients, we detected bi-allelic variants in *MMACHC* in 55 patients that involved 21 different types of reported pathogenic variants. The remaining patient carried a heterozygous variant in *MMACHC* and a *PRDX1* variant causing secondary epimutation of *MMACHC*. The most common variant in *MMACHC* was c.482G > A (36.6%), and it was followed by c.609G > A (16.1%). In agreement with this finding, Lerner-Ellis et al. also found that c.482G > A was the most frequent variant in their population of late-onset cases, but the c.609G > A variant was not common in their population of late-onset cases [18]. c.609G > A is the most common variant in cblC-deficient patients in China [3, 5, 19]. In our previous study, variable phenotypes and outcomes associated with the *MMACHC* c.609G > A homologous mutation in 149 Chinese patients were observed. 101 (76.5%) cases had early-onset disease and 31 (23.5%) had late-onset disease [2]. However, in the patients of this study, the heterozygous c.609G > A variant was detected along with another variant in the *MMACHC* gene. Further, the c.658_660delAAG variant was the third and the c.80A > G variant was the fourth most common variant in this study. These variants have been reported in other studies too [15, 16].

Neuropsychiatric diseases are frequent in patients with c.482G > A or c.609G > A variants [2, 18, 19]. In 13 patients in the present cohort with spastic paraplegia, 11 (84.6%) had a c.482G > A variant. This finding suggests that the c.482G > A variant may be the most common variant in late-onset patients with neuropsychiatric presentations [18]. Six patients with the c.80A > G and c.609G > A compound heterozygotic variants displayed neuropsychiatric symptoms or pulmonary hypertension. These results suggest that diseases of the cardiovascular system should be considered in patients with a c.80A > G variant. This finding is supported by previous studies [17, 20, 21].

In this study, seven (6.3%) patients with a heterozygous c.567dupT variant presented with neuropsychiatric diseases during adolescence. Previously, c.567dupT has been detected in early-onset patients with hydrocephalus secondary to cblC deficiency [22]. c.567dupT has also been found in two alleles of 26 late-onset patients in another study [5]. The c.394C > T variant was detected in four (3.6%) cases of this cohort. Lerner-Ellis et al. reported 42 different variants in 204 patients, and c.394C > T was detected in 34 alleles [20]. Further, it has been reported that individuals with c.394C > T tend to present with late-onset disease [18]. Morel et al. studied phenotype-genotype correlations in 37 patients from published case reports and found that the c.394C > T variant is common in Asiatic-Indian/Pakistani/Middle Eastern populations. In their study, 9 out of 12 late-onset cases presented with acute neurological symptoms. Four of these nine patients were homozygous for the c.394C > T variant, and two showed compound heterozygosity for the c.271dupA and c.394C > T variants [21]. Thus, c.567dupT and c.394C > T in this study mainly related to neuropsychiatric diseases during adolescence.

In one case (P56) of the present cohort, only one heterozygous c.609G > A in the *MMACHC* gene was identified. Significantly elevated blood tHcy, propionyl-carnitine, and urine methylmalonic acid are indicative of cblC deficiency. A c.515-48_515-47insTTA variant of unknown pathogenicity in the *PRDX1* gene, which was reported to cause *MMACHC* hypermethylation [23], was also found. This variant might have been associated with cb1C deficiency in this patient.

In the present study, all 56 patients were considered healthy before onset of the disease. Most cases had metabolic disturbances during the acute phase of the disease. Markedly increasing of blood propionylcarnitine, tHCY (10–14 fold), and urine methylmalonic acid were observed in most of the patients. High doses of cobalamin, l-carnitine, and betaine are administered in the acute phase to reduce the blood level of tHCY and correct the metabolic status as soon as possible [11, 24, 25]. Patients tend to regain their ability to learn and walk as the levels of their metabolic markers decrease. In this study, the problems related to the cardiovascular and pulmonary systems of the patients were reversed with this treatment regimen. Following treatment and improvement, patients gradually returned to school and started to live normal lives. Nine patients graduated and are working, and two female patients are married and have had healthy babies [13]. These results show that with appropriate treatment, patients with cblC deficiency can live normal lives [5, 26, 27].

## Conclusions

Patients with late-onset cb1C deficiency present with a wide variety of nonspecific clinical features that can easily be misdiagnosed as other conditions. The findings demonstrate that physicians can determine the accurate diagnosis in such patients with biochemical and genetic analyses. Further, after metabolic treatment, most patients can fully recover and live a normal life. These results indicate that differential diagnosis of inherited metabolic disorders should be considered for previously healthy adolescent patients who present with neuropsychiatric diseases and multiple organ damage.

## Methods

### Patients

This study included 56 Chinese patients with late-onset cb1C deficiency who were diagnosed and followed up at Peking University First Hospital between 2002 and May 2021. Their diagnosis was confirmed by biochemical and genetic analyses.

### Routine examination

Body weight, height, and secondary sex characteristics were recorded to evaluate the growth and sexual development of the patients. Blood pressure, electrocardiography, and echocardiography were used for cardiovascular monitoring. Routine examinations of blood, urine, glucose, insulin, and hepatic and renal functions were conducted in all the patients. Bone density and serum vitamin D were also measured. All the patients underwent cranial magnetic resonance imaging or computed tomography.

### Biochemical assays

Amino acids, free carnitine, and acyl-carnitines in dried blood spots were analyzed by liquid chromatography-tandem mass spectrometry (API 3200, Triple Quad 4500; Applied Biosystems, CA, USA). The concentrations of the metabolites were calculated automatically using the Chemoview software [2, 28].

Gas chromatography and mass spectrometry (GC/MS) was performed with GCMS-QP 2010 (Shimadzu Corporation, Kyoto, Japan) to analyze urine organic acids, according to a previously established protocol [28–30]. Data were collected using the GC/MS solution software. Plasma tHCY was detected by chemiluminescence immunoassay (Abbott I2000, USA).

### Genetic analysis

Peripheral blood samples were collected from the patients and their parents. DNA was extracted using a DNA Isolation Kit (AU1802; Bioteke, China). Purified DNA samples were sent to Running Gene Inc. (Beijing, China) or Berry Genomics Corporation (Beijing, China) for next-generation sequencing to screen variants in patients. Each variant was evaluated according to the Human Gene Mutation Database (HGMD, https://my.qiagendigitalinsights.com/bbp/view/hgmd/pro/gene.php?gene=MMACHC) and ClinVar (https://www.ncbi.nlm.nih.gov/clinvar/?term=MMACHC%5Bgene%5D&redir=gene).

### Treatment

For patients in the acute decompensation stage, initial therapy included adenosylcobalamin (3 mg/day) or hydroxycobalamin (10 mg/day) administered intramuscularly, l-carnitine (2–3 g/day), intravenous fluid therapy with glucose and electrolytes, oral betaine (3–9 g/day), folate (5–15 mg/day), and symptomatic treatment. After their condition stabilized, the dosages were reduced. Individual long-term metabolic treatment was adjusted according to their clinical condition [11, 24, 26].

## Supplementary Information

**Additional file 1.** Detailed clinical information for each enrolled subject.

## Data Availability

All data generated or analyzed during this study are included in this published article.

## Abbreviations

MMA: Methylmalonic aciduria
cblC: Cobalamin C
LC–MS/MS: Liquid chromatography-tandem mass spectrometry
GC/MS: Gas chromatography and mass spectrometry
tHCY: Total plasma homocysteine
MRI: Magnetic resonance imaging
CT: Computerized tomography
MMACHC: Cytoplasmic chaperone protein methylmalonic aciduria and homocystinuria
